# A simple, rapid, low-cost technique for naked-eye detection of urine-isolated TMPRSS2:ERG gene fusion RNA

**DOI:** 10.1038/srep30722

**Published:** 2016-07-29

**Authors:** Kevin M. Koo, Eugene J. H. Wee, Paul N. Mainwaring, Matt Trau

**Affiliations:** 1 Centre for Personalized Nanomedicine, Australian Institute for Bioengineering and Nanotechnology (AIBN), The University of Queensland, QLD 4072, Australia; 2 School of Chemistry and Molecular Biosciences, The University of Queensland, QLD 4072, Australia

## Abstract

The TMPRSS2:ERG gene fusion is one of a series of highly promising prostate cancer (PCa) biomarker alternatives to the controversial serum PSA. Current methods for detecting TMPRSS2:ERG are limited in terms of long processing time, high cost and the need for specialized equipment. Thus, there is an unmet need for less complex, faster, and cheaper methods to enable gene fusion detection in the clinic. We describe herein a simple, rapid and inexpensive assay which combines robust isothermal amplification technique with a novel visualization method for evaluating urinary TMPRSS2:ERG status at less than USD 5 and with minimal equipment. The assay is sensitive, and rapidly detects as low as 10^5^ copies of TMPRSS2:ERG transcripts while maintaining high levels of specificity.

Current prostate cancer (PCa) screening relies mainly on measuring serum prostate-specific antigen (PSA) levels which has clearly demonstrated improvements in patient survival^1^,^2^,^3^. Although PSA screening has been utilized widely for several decades, its value as a biomarker is controversial due to its poor sensitivity and specificity. As a consequence, this has frequently led to over diagnosis and unnecessary medical expenses^1^,^4^,^5^. Therefore, this has encouraged researchers to develop a new generation of more accurate PCa screening biomarkers. In 2005, the gene fusion between *transmembrane protease, serine 2* (TMPRSS2) and *v-ets avian erythroblastosis virus E26 oncogene homolog* (ERG) was reported as a recurring event in PCa^6^. Further studies have confirmed TMPRSS2:ERG to be a highly-specific PCa biomarker that is present in at least 50% of PCa cases^7^ and is associated with PCa progression through inducing androgen-regulated ERG overexpression as well as partnering with other oncogenic events such as PTEN loss^8^. The prognostic potential of the TMPRSS2:ERG biomarker has been demonstrated in several studies in which TMPRSS2:ERG presence was associated with more aggressive forms of PCa and poorer clinical prognosis (higher PSA level, Gleason score and/or tumour stage)^9^,^10^. Additionally, TMPRSS2:ERG transcripts are also detectable in urine as a potential non-invasive and convenient biomarker for early PCa-detection. Most importantly, TMPRSS2:ERG is a binary biomarker which is commonly associated with premalignant PCa but not benign conditions, thus making it the most specific PCa biomarker to date^11^. For treatment purposes, recent studies have shown TMPRSS2:ERG to be a potential drug target for impeding tumor growth and metastasis^12^,^13^. Therefore, the detection of TMPRSS2:ERG presents as an unique opportunity for developing novel binary (positive/negative) strategies for PCa screening or treatment.

Reverse transcription-polymerase chain reaction (RT-PCR), and fluorescence *in situ* hybridization (FISH) are the most commonly-used techniques for TMPRSS2:ERG detection in clinical PCa specimens. While FISH assays are useful for detecting gene fusions on both DNA and RNA in tissue cells^14^, they have limited sensitivity due to high background fluorescence. As a consequence, FISH may be unsuitable for detecting trace amounts of TMPRSS2:ERG transcripts in clinical samples such as urine specimens. In contrast, RT-PCR is a sensitive assay which is routinely used in many studies for detecting low levels of TMPRSS2:ERG transcripts in PCa urine specimens^15^,^16^,^17^. However, PCR techniques are constrained to laboratory settings by the high number of thermal cycles needed to detect low TMPRSS2:ERG levels (typically requiring ≥ 120 min); need for highly trained personnel; and required specialized equipment. Recently, an isothermal transcription-mediated amplification (TMA)-based assay^18^ was developed to resolve issues faced by RT-PCR methods but the chemiluminescence readout of this assay still has limitations such as high reagent cost, prolonged experimental procedures as well as the need for specialized instrumentation. Hence, it is still of interest to the community to develop a rapid yet inexpensive novel point-of-care assay to address the shortcomings of current methodologies for routine TMPRSS2:ERG screening.

Colorimetric assays based on gold nanoparticles aggregation have been developed for sensitive and rapid detection of polynucleotide sequences^19^. However, the subtle colour changes of these assays frequently require the use of spectrophotometry for readout. Therefore, an opportunity presents itself to enable easier visual detection by naked-eye interpretation; and one such possibility could be polymer (eg. DNA)-mediated flocculation assays^20^,^21^,^22^ which typically possess a binary threshold for flocculation output and interpretation^23^,^24^,^25^. This sort of binary evaluation system would also complement the binary nature of the TMPRSS2:ERG biomarker.

Herein, we present a novel TMPRSS2:ERG detection technique by combining a robust isothermal amplification method with a flocculation-based visual readout. The assay is a relatively simple, rapid, and inexpensive methodology for evaluating urinary TMPRSS2:ERG status with good sensitivity and specificity.

## Results

### Assay principle

**Figure 1:**
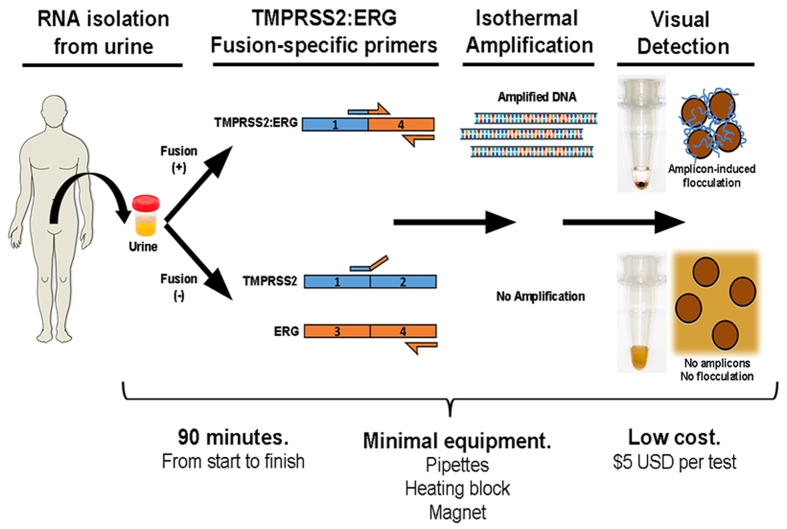
Schematic representation of rapid and simple assay for TMPRSS2:ERG gene fusion detection in prostate cancer urine specimens. Total RNA which potentially include TMPRSS2:ERG transcripts is first isolated from the urine specimen of a screening candidate. Isothermal reverse transcriptase-recombinase polymerase amplification is used to generate cDNA amplicons exclusively in the presence of TMPRSS2:ERG fusion transcripts. This is followed by adding SPRI magnetic beads to spontaneously bind post-amplification sequences. Successful amplicons (i.e. TMPRSS2:ERG-positive) induces bridging flocculation of magnetic beads to produce a colourless solution. Specimens without TMPRSS2:ERG transcripts result in no amplification and beads bound with only primer sequences are of insufficient length to mediate flocculation, thereby producing a brown-coloured solution indicative of a negative TMPRSS2:ERG reading.

Figure 1 depicts the working principle of our assay. This assay first employed magnetic beads to isolate and purify total RNA from urinary sediments by the Solid Phase Reversible Immobilization (SPRI) technique^26^. Next, isothermal reverse transcription-recombinase polymerase amplification^27^ (RT-RPA) was utilized to rapidly amplify TMPRSS2:ERG transcripts within 30 min. Lastly, amplified DNA (i.e. positive for TMPRSS2:ERG) were detected using a DNA-mediated bridging flocculation phenomenon whereby only amplicons of sufficient length (*i.e*., ≥200 bp)^22^ and amounts could initiate flocculation of magnetic beads to maintain a colourless solution.

### Assay specificity for TMPRSS2:ERG

**Figure 2:**
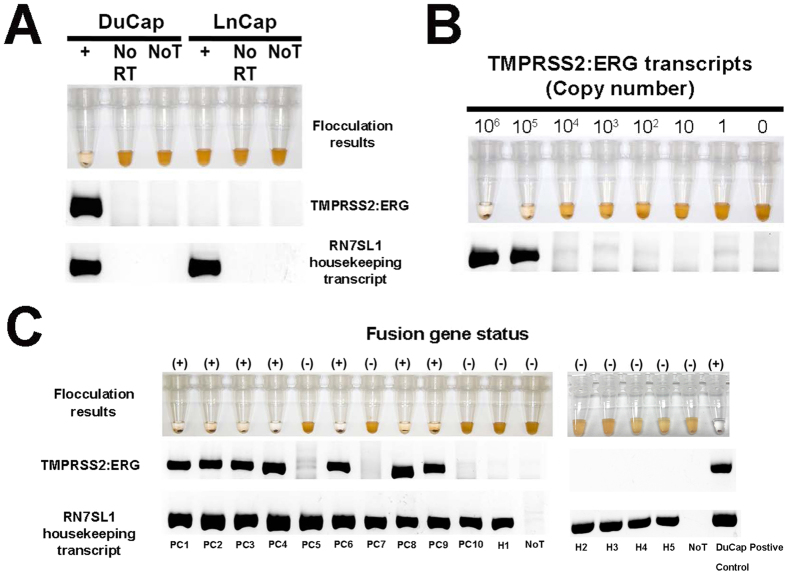
Detection of TMPRSS2:ERG transcripts in prostate cancer cell lines and clinical urine specimens. (**A**) TMPRSS2:ERG detection in DuCap and LnCap cell lines. (**B**) Titration assay of spiking different concentrations of synthetic TMPRSS2:ERG RNA into LnCap RNA background. (**C**) Two separate runs of TMPRSS2:ERG detection in urine specimens of 10 castrate-resistant prostate cancer patients and 5 healthy males with no prior prostate cancer history; 1st run (Left): PC1-PC10 & H1; 2nd run (Right): H2-H5. No RT and NoT refers to no-reverse transcriptase and no-template controls respectively. Top row: Images of flocculation assays for RT-RPA reactions. Bottom: Gel electrophoresis images corresponding to the RT-RPA reactions. ***Note:** Gel electrophoresis experiments were all performed under similar experimental conditions (200* *V, 30* *min) and images have been cropped for clarity of presentation, full-length blots/gels are presented in* Supplementary Fig. 4.

As a proof-of-concept, we applied our method to detect the most common TMPRSS2:ERG fusion transcript (TMPRSS2 exon1 to ERG exon 4) in cultured DuCap (TMPRSS2:ERG-positive) and LnCap (TMPRSS2:ERG-negative) human PCa cells^6^. The RT-RPA primers spanned the fusion junction of TMPRSS2 and ERG (Fig. 1), thus reducing the likelihood of amplifying wild type transcripts. As expected, DuCap cells were tested positive (colourless) for TMPRSS2:ERG while LnCap were negative (brown-coloured) (Fig. 2A). The amplified products were verified by agarose gel electrophoresis which only showed a 216 bp product band for the DuCap cell line but not for the LnCap cell line. When cell line RNA (NoT control) and reverse transcriptase (no RT control) were omitted from the RPA reaction, no products were detected to affirm that products were TMPRSS2:ERG RNA-dependent. To control for RNA loading, the housekeeping transcript, RN7SL1^28^, was used. We also confirmed the identity of the amplicons through sequencing (Supplementary Fig. 1). The successful detection of TMPRSS2:ERG in DuCap cells demonstrated the specificity of our assay to detect TMPRSS2:ERG transcripts from total RNA. In addition, the colorimetric readout of the assay clearly showed the distinct colour changes between TMPRSS2:ERG states, thus potentially allowing a rapid and convenient binary evaluation of gene fusion status by the naked-eye with minimal equipment.

### Assay limit-of-detection

We then tested the sensitivity of our assay by titrating known amounts of synthetic TMPRSS2:ERG RNA into 10 ng of LnCap total RNA background (for each titation). As little as 10^5^ copies of TMPRSS2:ERG RNA (Fig. 2B) could be detected and this number of fusion transcripts was approximately equivalent to that of a single-cell^29^.

### Urine TMPRSS2:ERG in PCa patients

To demonstrate clinical utility, we then applied our assay to analyze TMPRSS2:ERG status in clinical urine specimens from 10 metastatic castration-resistant PCa (CRPCa) patients and 5 healthy control patients. Using RN7SL1 housekeeping transcripts as input control, we detected TMPRSS2:ERG transcripts from urine specimens in 7 out of 10 metastatic CRPC patients (70%) but not in the healthy patient and no-template (NoT) controls (Fig. 2C). These outcomes were further validated by the current gold standard RT-PCR assay (Supplementary Fig. 2) with a different pair of primers (Table 1). Both our assay and RT-PCR gave identical results, thus supporting our TMPRSS2:ERG assay as a feasible faster alternative to RT-PCR.

## Discussion

The current use of PSA levels for PCa diagnosis has led to incidences of false-positive/negative results^30^. Gene fusions are one of the new generation of promising PCa biomarkers which could replace or supplement the PSA test as it is highly PCa-specific and absent in benign conditions^7^,^9^. Thus, we hypothesized that an assay which is able to reliably and rapidly detect gene fusion presence at a low-cost, could be a useful tool for diagnosing PCa in the point-of-care setting. Here we report a novel urine-based gene fusion assay by detecting the common TMPRSS2 (exon 1):ERG (exon 4)^6^ in PCa patients. This assay differs from previous reports of gene fusion detection methodologies in the literature as it is able to give a positive/negative flocculation-based outcome detectable by the naked-eye within 90 minutes with minimal equipment.

The bridging flocculation phenomenon in our assay refers to the ability of RT-RPA amplicons to initiate cross-linking of magnetic beads and flocculate out of an aqueous dispersion, thus rendering the solution colourless (Fig. 1). The length of the amplicons limits this flocculation mechanism as short-length primers (in the event of unsuccessful amplification) are unable to induce bridging of magnetic beads. Bridging flocculation of magnetic beads is particularly useful for visual detection of a variety of nucleic acid-based pathogens after successful amplification^20^,^22^. Whilst various lines of research have reported visual DNA detection through magnetic particles aggregation^31^,^32^,^33^, our assay is unique in requiring only a conventional magnetic plate (i.e. static magnetic field) after a rapid 30 min isothermal amplification.

Importantly, the sensitivity (10^5^ TMPRSS2:ERG transcript copies) and specificity of this approach (Fig. 2A,B) could be useful for detecting low TMPRSS2:ERG amount in a complex clinical sample such as urine. Furthermore, the colorimetric readout of the assay clearly demonstrated the distinct colour changes between TMPRSS2:ERG states, thus potentially allowing a rapid and convenient binary evaluation of gene fusion status. Unlike RT-PCR/FISH assays which require time-consuming preparation, long assay times, and costly fluorescence readouts; our assay is able to avoid these complications. Firstly, the RT-RPA technique is readily-available commercially in pellet form and preparation steps are simple, quick and minimal. Secondly, the amplification process is isothermal and rapid to facilitate shorter assay turn-around-time. In contrast to the traditional fluorescence readouts of RT-PCR/FISH; our novel flocculation-based colorimetric readout is simpler and quicker to prepare, more cost-effective without need for specialized instruments, and free from conventional fluorescence bleaching issues.

To demonstrate potential clinical utility, we used our assay to investigate the TMPRSS2:ERG status in urine samples from 10 CRPCa patients and 5 healthy controls (Fig. 2C). The results were subsequently verified with gold standard RT-PCR with 100% concordance (Supplementary Fig. 2). Furthermore, our assay findings were also consistent with literature reports describing the presence of TMPRSS2:ERG in late-stage metastatic CRPCa patients^34^. It is also worth highlighting that the assay results were obtained in approximately 90 minutes, and we have also been able to detect TMPRSS2:ERG transcripts from whole urine specimens without any need for ultracentrifugation to concentrate urinary sediments (Supplementary Fig. 3). However, we found that detection from whole urine is only possible if the assay is performed immediately (within 2 hours) after specimen collection. We hypothesized that RNA from whole urine most likely degraded with time, therefore decreasing RT-RPA efficiency. In contrast, sediment RNA remained stable for extraction even after 12 hrs (Supplementary Fig. 3). While using whole urine may be convenient, due to logistic limitations and assay reproducibility, urinary sediments are the more viable RNA source for this study. Nonetheless, if the assay could be done immediately on-site, detecting TMPRSS2:ERG from whole urine may still be a possibility and thus further simplifying the method.

There are several aspects of our assay which could be further developed and improved upon in order to realize its full potential as a clinical tool. First, the flocculation colorimetric readout is not quantitative and only offers a discrete yes/no result in its current format. However, this could be overcome by designing softwares to measure the intensity and size of cross-linked magnetic beads. As more transcripts could lead to higher flocculation, the intensity and size of the flocculated magnetic beads could be quantitative for TMPRSS2:ERG transcripts. Furthermore, our assay could be modified as a chip-based assay in the future as isothermal amplification, magnetic beads, and visual readout are components which are ideal for chip-based assay development. By integrating our assay onto a chip, we could potentially increase assay throughput and multiplexibility, improve portability through assay miniaturization, and eventually automate the whole assay process. Nonetheless, the current assay is still an attractive low resource alternative as compared to RT-PCR or FISH.

In conclusion, we describe a novel coupling of isothermal RT-RPA with a flocculation readout to enable a rapid, convenient, inexpensive and non-invasive method for detecting PCa gene fusion RNA. To our knowledge, our colorimetric readout method is the first display of using the RT-RPA technique and bridging flocculation phenomena to evaluate urinary gene fusion transcripts such as TMPRSS2:ERG. This positive/negative readout is easily evaluated by the naked-eye and also complements binary biomarkers such as gene fusions. We envisage that our assay may have wide application potential in detecting different gene fusion occurrences in other forms of cancers or diseases by simply tailoring primer sequences.

## Methods

### RNA extraction from cell lines

The cell lines DuCap and LnCap were generously donated by Matthias Nees (VTT, Finland); Gregor Tevz (APCRC, Australia); and Michelle Hill (UQDI, Australia). The cells were cultured in RPMI-1640 growth media (Life Technologies, Australia) supplemented with 10% fetal bovine serum (Life Technologies, Australia) in a humidified incubator containing 5% CO_2_ at 37 °C. RNA was extracted using Trizol reagent (Life Technologies, Australia) and RNA integrity and purity were checked using a NanoDrop Spectrophotometer (Thermo Scientific, USA).

### RNA extraction from clinical urinary specimens

Ethics approval was obtained from The University of Queensland Institutional Human Research Ethics Committee (Approval No. 2004000047). Methods pertaining to clinical samples were carried out in accordance with approved guidelines and informed consent was obtained from all subjects prior to sample collection.

Voided urine specimens were collected from 10 men undergoing treatment for CRPCa and 5 healthy men with no clinical history of PCa. Urine specimens (30–50 ml) were centrifuged at 700 *g* for 10 min and urinary sediments were washed with ice-cold PBS buffer before being centrifuged again at the same conditions. 25 μl of lysis buffer (150 mM Tris-HCI pH 8.0, 4.5 M guanidium-HCI, 3% v/v Triton-X and 1.5 mM EDTA) was added to 50 μl of PBS-suspended urinary sediments with vigorous mixing to release total RNA. Next, total RNA was purifed from 25 μl of cell lysate using the Agencourt AMPure XP kit (Beckman Coulter, USA). Briefly, two volumes SPRI reagent was first incubated with the cell lysate for 10 minutes. The RNA-bound magnetic beads were then separated from the lysate using a magnet and washed once with 100% isopropanol and twice more with 80% ethanol. Finally, total RNA was eluted in 25 μl of RNase-free water.

### cDNA synthesis and nucleic acid amplification

For cell line experiments, the TwistAmp Basic RPA kit (Twist-DX, UK) was used with slight modifications to manufacturer’s instructions. Briefly, 1 μl of extracted RNA, 50 units of MMuLV reverse transcriptase (New England Biolabs, UK) and 500 nM of each primer (Table 1) were added to make a 12.5 μl reaction volume prior to incubation at 39 °C for 20 min. For all other experiments, the TwistAmp Basic RT-RPA kit (Twist-DX, UK) was used with slight modifications to manufacturer’s instructions to simultaneously generate cDNA and rapidly amplify the cDNA templates in a single tube reaction. Briefly, 2 μl of extracted RNA and 500 nM of each primer (Table 1) were added to make a 12.5 μl reaction volume prior to incubation at 41 °C for 30 min.

### Bridging flocculation colorimetric readout

Amplicons were similarly purified using SPRI as previously described but with only one ethanol wash step. After purification, 5 μl of purified amplicons were incubated with 2 volumes of SPRI magnetic beads for 5 min before bead separation by magnet. Then, 20 μL of flocculation buffer (200 mM sodium acetate, pH 4.4) was added to the beads. After 1 min of incubation, the mixture was gently agitated using a magnet and observed for colour change.

### RT-PCR

RT-PCR was performed on extracted total RNA from clinical urine specimens to validate results of our assay. The KAPA SYBR FAST One-Step qRT-PCR kit (KAPA BIOSYSTEMS, USA) was used to set up a single reaction volume of 10 μl for each sample. Each reaction volume consist of 1X KAPA SYBR FAST qPCR Master Mix, 200 nM of each primer (Table 1), 1X KAPA RT Mix and 3 μl of extracted RNA. The tubes were incubated at 42 °C for 10 min to synthesize cDNA, followed by 95 °C for 5 min to deactivate RT before cycling 35 times (95 °C for 30 s, 70 °C for 30 s and 72 °C for 1 min) and finished with 72 °C for 10 min. Lastly, the RT-PCR products were visualized on 1.5% agarose gel to verify amplification.

## Additional Information

**How to cite this article**: Koo, K. M. *et al*. A simple, rapid, low-cost technique for naked-eye detection of urine-isolated TMPRSS2:ERG gene fusion RNA. *Sci. Rep*. **6**, 30722; doi: 10.1038/srep30722 (2016).

## Supplementary Material

Supplementary Information

## Figures and Tables

**Table 1:** Oligonucleotide sequences.

Oligonucletotides	Sequence (5′ to 3′)
RT-RPA Forward, RT-PCR Forward	CCTGGAGCGCGGCAGGAAGCCTTATCAGTTG
RT-RPA Reverse	GCTAGGGTTACATTCCATTTTGATGGTGAC
RT-PCR Reverse	TCCTGCTGAGGGACGCGTGGGCTCATCTTG

Oligonucleotides were purchased from Integrated DNA Technologies, USA.

## References

[b1] Schroder F. H. . Prostate-Cancer Mortality at 11 Years of Follow-up. N. Eng. J. Med. 366, 981–990 (2012). 10.1056/NEJMoa1113135 PMC6027585 22417251

[b2] Prensner J. R., Rubin M. A., Wei J. T. & Chinnaiyan A. M. Beyond PSA: The next generation of prostate cancer biomarkers. Science Transl. Med. 4, 127rv3 (2012). 10.1126/scitranslmed.3003180 PMC3799996 22461644

[b3] Cuzick J. . Prevention and early detection of prostate cancer. Lancet Oncol. 15, E484–E492 (2014). 25281467 10.1016/S1470-2045(14)70211-6 PMC4203149

[b4] Andriole G. L. . Prostate cancer screening in the randomized prostate, lung, colorectal, and ovarian cancer screening trial: Mortality results after 13 Years of follow-up. J. Natl. Cancer Inst. 104, 125–132 (2012). 22228146 10.1093/jnci/djr500 PMC3260132

[b5] Shoag J. . Efficacy of prostate-specific antigen screening: Use of regression discontinuity in the PLCO cancer screening trial. JAMA Oncol. 1, (2015). 10.1001/jamaoncol.2015.2993 26291583

[b6] Tomlins S. A. . Recurrent fusion of TMPRSS2 and ETS transcription factor genes in prostate cancer. Science 310, 644–648 (2005). 16254181 10.1126/science.1117679

[b7] Tomlins S. A. . ETS gene fusions in prostate cancer: From discovery to daily clinical practice. Eur. Urol. 56, 275–286 (2009). 19409690 10.1016/j.eururo.2009.04.036

[b8] Carver B. S. . Aberrant ERG expression cooperates with loss of PTEN to promote cancer progression in the prostate. Nat. Genet. 41, 619–624 (2009). 19396168 10.1038/ng.370 PMC2835150

[b9] Wang J. H., Cai Y., Ren C. X. & Ittmann M. Expression of variant TMPRSS2/ERG fusion messenger RNAs is associated with aggressive prostate cancer. Cancer Res. 66, 8347–8351 (2006). 16951141 10.1158/0008-5472.CAN-06-1966

[b10] Leyten G. . Prospective multicentre evaluation of PCA3 and TMPRSS2-ERG gene fusions as diagnostic and prognostic urinary biomarkers for prostate cancer. Eur. Urol. 65, 534–542 (2014). 23201468 10.1016/j.eururo.2012.11.014

[b11] Vaananen R.-M., Ochoa N. T., Bostrom P. J., Taimen P. & Pettersson K. Altered PCA3 and TMPRSS2-ERG expression in histologically benign regions of cancerous prostates: a systematic, quantitative mRNA analysis in five prostates. BMC Urol. 15, 88 (2015). 26294063 10.1186/s12894-015-0077-7 PMC4546243

[b12] Rahim S. & Uren A. Emergence of ETS transcription factors as diagnostic tools and therapeutic targets in prostate cancer. Am. J. Transl. Res. 5, 254–268 (2013). 23634237 PMC3633969

[b13] Rahim S. . A small molecule inhibitor of ETV1, YK-4-279, prevents prostate cancer growth and metastasis in a mouse xenograft model. PLoS One 9, e114260 (2014). 25479232 10.1371/journal.pone.0114260 PMC4257561

[b14] Yoshimoto M. . Three-color FISH analysis of TMPRSS2/ERG fusions in prostate cancer indicates that genomic microdeletion of chromosome 21 is associated with rearrangement. Neoplasia 8, 465–469 (2006). 16820092 10.1593/neo.06283 PMC1601467

[b15] Laxman B. . Noninvasive detection of TMPRSS2 : ERG fusion transcripts in the urine of men with prostate cancer. Neoplasia 8, 885–888 (2006). 17059688 10.1593/neo.06625 PMC1715928

[b16] Hessels D. . Detection of TMPRSS2-ERG fusion transcripts and prostate cancer antigen 3 in urinary sediments may improve diagnosis of prostate cancer. Clin. Cancer Res. 13, 5103–5108 (2007). 17785564 10.1158/1078-0432.CCR-07-0700

[b17] Laxman B. . A first-generation multiplex biomarker analysis of urine for the early detection of prostate cancer. Cancer Res. 68, 645–649 (2008). 18245462 10.1158/0008-5472.CAN-07-3224 PMC2998181

[b18] Tomlins S. A. . Urine TMPRSS2:ERG fusion transcript stratifies prostate cancer risk in men with elevated serum PSA. Science Transl. Med. 3, 94ra72 (2011). 10.1126/scitranslmed.3001970 PMC3245713 21813756

[b19] Rosi N. L. & Mirkin C. A. Nanostructures in biodiagnostics. Chem. Rev. 105, 1547–1562 (2005). 15826019 10.1021/cr030067f

[b20] Wee E. J. H., Lau H. Y., Botella J. R. & Trau M. Re-purposing bridging flocculation for on-site, rapid, qualitative DNA detection in resource-poor settings. Chem. Commun. 51, 5828–5831 (2015). 10.1039/c4cc10068a 25622026

[b21] Wee E. J. H., Thu Ha N. & Trau M. A simple bridging flocculation assay for rapid, sensitive and stringent detection of gene specific DNA methylation. Sci. Rep. 5, 15028 (2015). 26458746 10.1038/srep15028 PMC4602207

[b22] Ng B. Y. C., Wee E. J. H., West N. P. & Trau M. Rapid DNA detection of Mycobacterium tuberculosis-towards single cell sensitivity in point-of-care diagnosis. Sci. Rep. 5, 15027 (2015).

[b23] Huggins M. L. Solutions of long chain compounds. J. Chem. Phys. 9, 440–440 (1941).

[b24] Flory P. I. Thermodynamics of high polymer solutions. J. Chem. Phys. 10, 51–61 (1942).

[b25] Healy T. W. & La Mer V. K. The energetics of flocculation and redispersion by polymers. J. Colloid Sci. 19, 323–332 (1964).

[b26] Deangelis M. M., Wang D. G. & Hawkins T. L. Solid-phase reversible immobilization for the isolation of PCR products. Nucleic Acids Res. 23, 4742–4743 (1995). 8524672 10.1093/nar/23.22.4742 PMC307455

[b27] Piepenburg O., Williams C. H., Stemple D. L. & Armes N. A. DNA detection using recombination proteins. Plos Biol. 4, 1115–1121 (2006). 10.1371/journal.pbio.0040204 PMC1475771 16756388

[b28] Galiveti C. R., Rozhdestvensky T. S., Brosius J., Lehrach H. & Konthur Z. Application of housekeeping npcRNAs for quantitative expression analysis of human transcriptome by real-time PCR. RNA 16, 450–461 (2010). 20040593 10.1261/rna.1755810 PMC2811673

[b29] Velculescu V. E. . Analysis of human transcriptomes. Nat. Genet. 23, 387–388 (1999). 10581018 10.1038/70487

[b30] Thompson I. M. . Prevalence of prostate cancer among men with a prostate-specific antigen level <= 4.0 ng per milliliter. *N*. Eng. J. Med. 350, 2239–2246 (2004). 10.1056/NEJMoa031918 15163773

[b31] Leslie D. C. . New detection modality for label-free quantification of DNA in biological samples via superparamagnetic bead aggregation. J. Am. Chem. Soc. 134, 5689–5696 (2012). 22423674 10.1021/ja300839n PMC3339050

[b32] Li J. . Label-free DNA quantification via a ‘pipette, aggregate and blot’ (PAB) approach with magnetic silica particles on filter paper. Lab Chip 13, 955–961 (2013). 23338039 10.1039/c2lc40975e

[b33] Lin C., Zhang Y., Zhou X., Yao B. & Fang Q. Naked-eye detection of nucleic acids through rolling circle amplification and magnetic particle mediated aggregation. Biosens. Bioelectron. 47, 515–519 (2013). 23643944 10.1016/j.bios.2013.03.056

[b34] Cai C. M., Wang H. Y., Xu Y. Y., Chen S. Y. & Balk S. P. Reactivation of androgen receptor-regulated TMPRSS2:ERG gene expression in castration-resistant prostate cancer. Cancer Res. 69, 6027–6032 (2009). 19584279 10.1158/0008-5472.CAN-09-0395 PMC2859723

