# Supplementary material for: A simple, rapid, low-cost technique for naked-eye detection of urine-isolated TMPRSS2:ERG gene fusion RNA
Source: Sci Rep. 2016 Jul 29;6:30722. doi: 10.1038/srep30722 (PMC4965811; doi:10.1038/srep30722)
Supplement: Supplementary Information [file srep30722-s1.pdf]

# A simple, rapid, low-cost technique for naked-eye detection of urine-isolated TMPRSS2:ERG gene fusion RNA

Kevin M. Koo, Eugene J. H. Wee, Paul N. Mainwaring, Matt Trau

| | |
|---|---|
| Supplementary Figure 1 | DNA sequencing of DuCap RNA amplicons after RT-RPA |
| Supplementary Figure 2 | RT-PCR of extracted RNA from patient urine specimens |
| Supplementary Figure 3 | Comparison of RNA stability in whole urine and urinary sediments |
| Supplementary Figure 4 | Full-length gel images of Figure 2 results |

**Supplementary Figure 1**

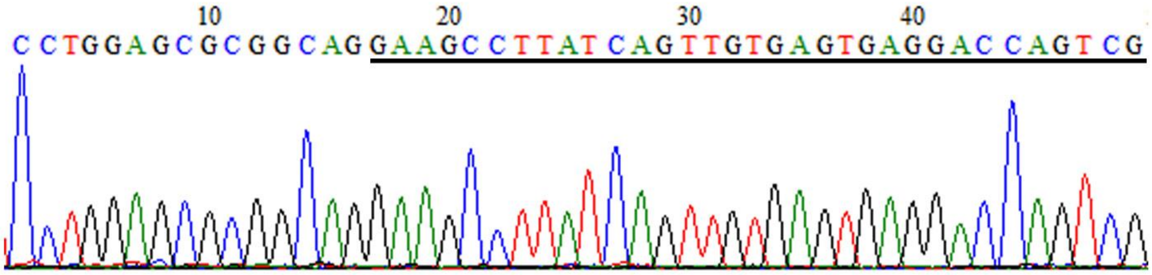


**Supplementary Figure 1.** DNA sequencing of DuCap RNA amplicons after RT-RPA. RT-RPA amplicons of extracted DuCap RNA was purified using SPRI magnetic beads and sequenced to verify amplification of target TMPRSS2:ERG region. *The ERG sequence bases are <u>underlined</u> to indicate the TMPRSS2:ERG fusion junction.

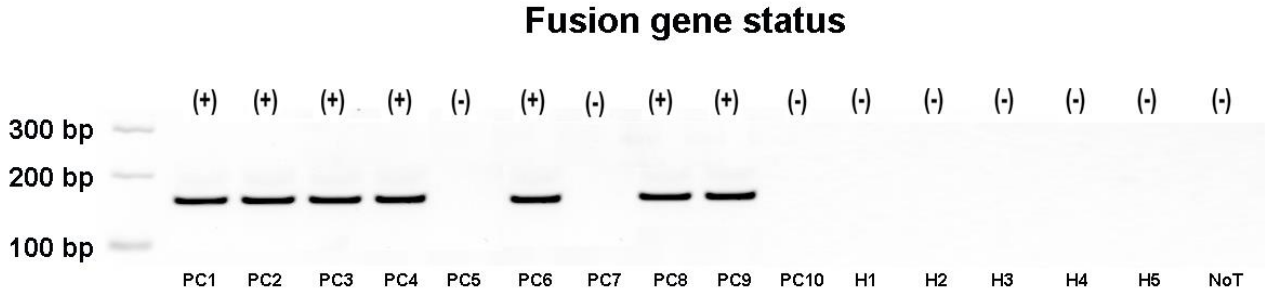


**Supplementary Figure 2.** RT-PCR of extracted RNA from patient urine specimens. Extracted RNA from urine specimens of 10 metastatic castrate-resistant prostate cancer patients and 5 healthy patients were amplified using RT-PCR for TMPRSS2:ERG detection. The RT-PCR amplicons were visualized on agarose gel and used to validate the screening results of our assay on the same group of patients (**Figure 2C**).

**Supplementary Figure 3**

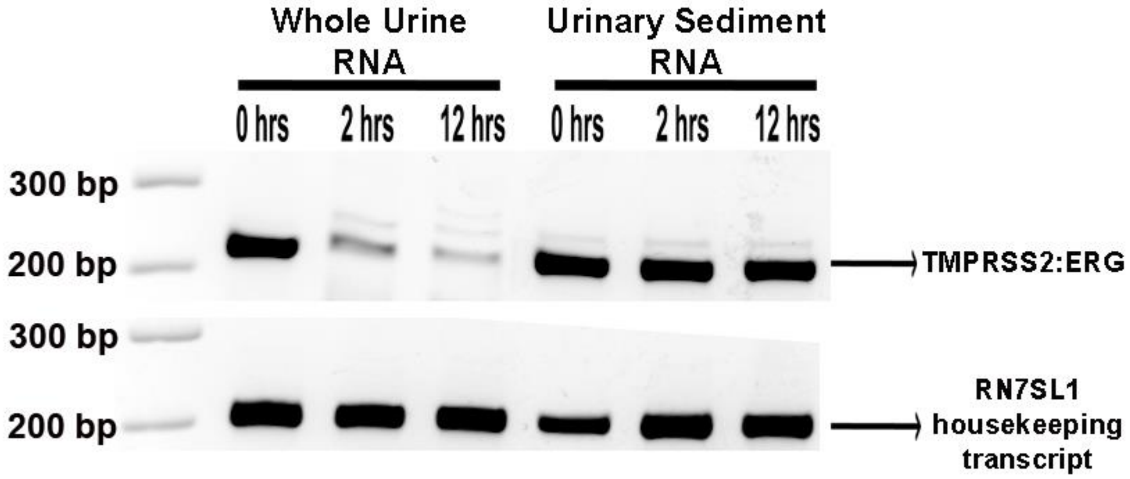


**Supplementary Figure 3.** Comparison of RNA stability in whole urine and urinary sediments. RT-RPA of patient whole urine and urinary sediment RNA extracted at 0, 2 and 12 hrs after specimen collection.

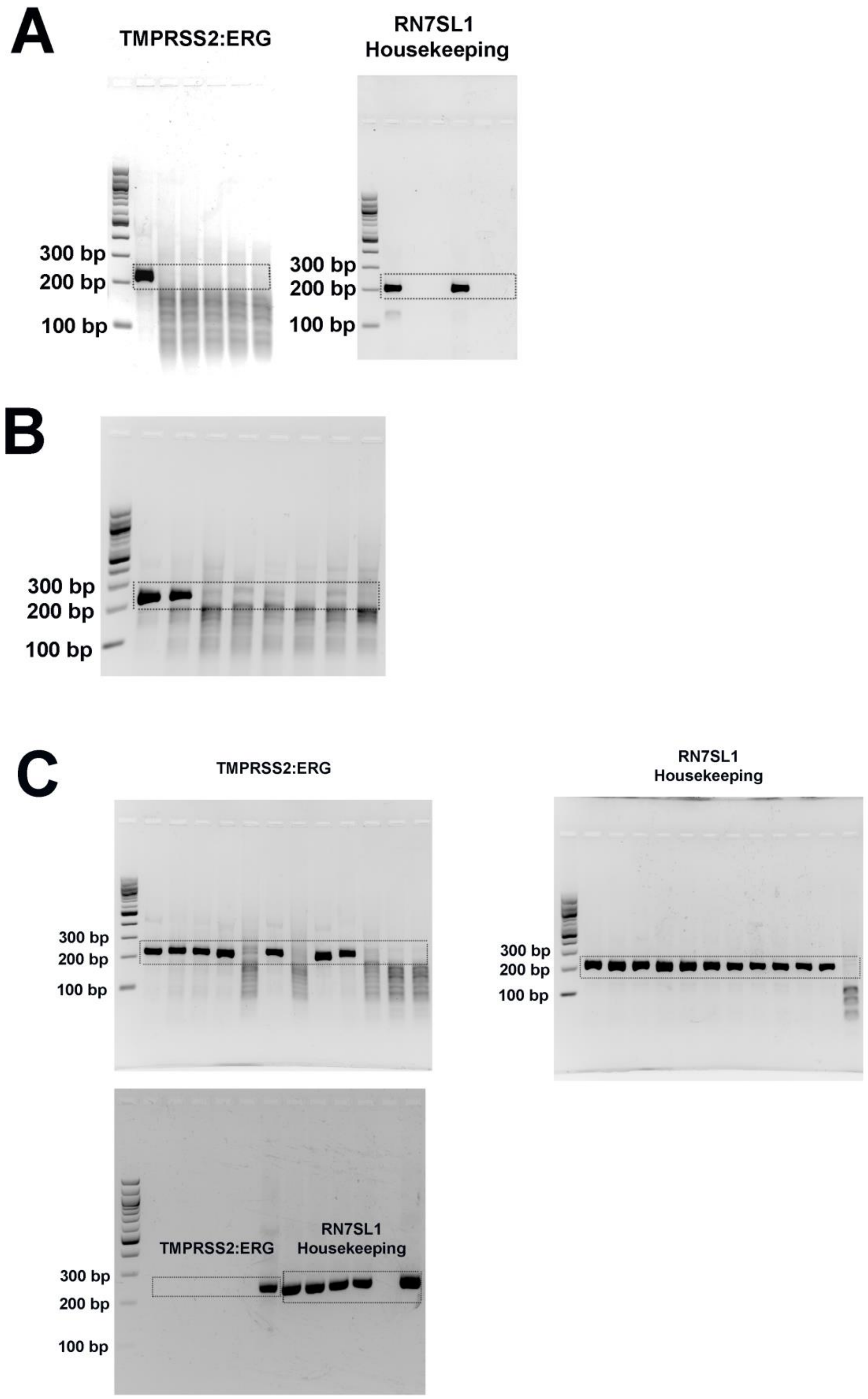


**Supplementary Figure 4.** Full-length gel images of Figure 2 results. Dotted outlines represent cropping lines of images shown in Figure 2A, B, and C.
